# Introducing a single-cell-derived human mesenchymal stem cell line expressing hTERT after lentiviral gene transfer

**DOI:** 10.1111/j.1582-4934.2008.00299.x

**Published:** 2008-03-04

**Authors:** Wolfgang Böcker, Zhanhai Yin, Inga Drosse, Florian Haasters, Oliver Rossmann, Matthias Wierer, Cvetan Popov, Melanie Locher, Wolf Mutschler, Denitsa Docheva, Matthias Schieker

**Affiliations:** a Experimental Surgery and Regenerative Medicine, Department of Surgery, Ludwig-Maximilians-University (LMU)Nussbaumstrafie 20, 80336 Munich, Germany; b Department of Orthopaedics, First Affiliated Hospital, School of Medicine, Xi'an Jiaotong University710061 Xi'an, Shaanx Province, P. R. China; c Center for Human Genetics and Laboratory MedicineLochhammerstr. 29, 82152 Munich, Germany

**Keywords:** mesenchymal stem cells, telomerase reverse transcriptase, gene transfer techniques, lentivirus

## Abstract

Human mesenchymal stem cells (hMSCs) can be readily isolated from bone marrow and differentiate into multiple tissues, making them a promising target for future cell and gene therapy applications. The low frequency of hMSCs in bone marrow necessitates their isolation and expansion *in vitro* prior to clinical use, but due to senescence-associated growth arrest during culture, limited cell numbers can be generated. The lifespan of hMSCs has been extended by ectopic expression of human telomerase reverse transcriptase (hTERT) using retroviral vectors. Since malignant transformation was observed in hMSCs and retroviral vectors cause insertional mutagenesis, we ectopically expressed hTERT using lentiviral gene transfer. Single-cell-derived hMSC clones expressing hTERT did not show malignant transformation *in vitro* and *in vivo* after extended culture periods. There were no changes observed in the expression of tumour suppressor genes and karyotype. Cultured hMSCs lack telomerase activity, but it was significantly increased by ectopic expression of hTERT. HTERT expression prevented hMSC senescence and the cells showed significantly higher and unlimited proliferation capacity. Even after an extended culture period, hMSCs expressing hTERT preserved their stem cells character as shown by osteogenic, adipogenic and chon-drogenic differentiation. In summary, extending the lifespan of human mesenchymal stem cells by ectopic expression of hTERT using lentiviral gene transfer may be an attractive and safe way to generate appropriate cell numbers for cell and gene therapy applications.

## Introduction

Human bone marrow-derived mesenchymal stem cells (hMSCs) are an attractive target for therapeutic cell transplantation, since they have a high proliferation capacity and maintain *in vitro* the ability to differentiate into a variety of mesenchymal tissues such as bone, cartilage, fat and muscle [1, 2]. However, for *in vivo* applications large cell numbers are needed. MSCs are readily collected from human bone marrow and expanded *in vitro*, but only 0.001–0.01% of nucleated cells are MSCs. The low frequency of hMSCs in bone marrow necessitates their isolation and expansion *in vitro* prior to clinical use. Yet, studies have shown that hMSCs undergo senescence-associated growth arrest under current culture conditions, a phenomenon termed replicative senescence [3]. HMSCs can achieve a maximum of 24–40 population doublings (PDs) *in vitro* before they lose their proliferation potential, homing and differentiation capacity [4–6].

Compelling evidence has been obtained by showing that telom-ere shortening is an important mechanism limiting the life span of normal human somatic cells in culture including MSCs [7, 8]. Telomere length is usually maintained by telomerase, a ribonuclear protein complex consisting of an integral RNA, which serves as telomeric template, and a catalytic subunit (TERT) with reverse transcriptase activity. In the absence of TERT, telomeres shorten during cell division, resulting in cell senescence and growth arrest. Since adult hMSCs lack telomerase activity *in vitro*[9–11], several groups have tried to overcome this hurdle by introducing the gene coding for the human TERT (hTERT) under the control of a constitutive promoter into MSCs. In hMSCs, ectopic expression of hTERT has abolished the senescence-associated phenotype and maintained MSC function including unlimited proliferation capacity, ability to differentiate into multiple cell lineages *in vitro* and *in vivo*[10, 12–14].

The excitement to have an unlimited cell source of hMSCs for therapeutic cell transplantation by ectopic expression of hTERT was limited by more recent finding that these cells underwent neoplastic transformation [15, 16]. A strong link between telomerase expression and many cancer types has been observed. Yet, telomerase is the critical enzyme in overcoming growth limitations due to telom-ere dysfunction, but does not cause growth deregulation [17]. There are, in fact, dozens of normal cell types that have been immortalized with telomerase without signs of malignant transformation, without altering pre-existing genetic abnormalities, and without altering differentiation capacity [17]. All hTERT-expressing hMSCs, which showed malignant transformation so far, used gamma-retroviral vectors. Insertional mutagenesis has been well established with these viruses and causes major concern when using lentiviral vectors due to their high similarity. However, no adverse events have been reported so far upon transplantation of cells transduced with lentiviral vectors. Here, we show for the first time that an MSC line generated by ectopic expressing of hTERT using lentiviral gene transfer did not cause malignant transformation and, therefore, may be a safe tool for *ex vivo* cell and gene therapy.

## Material and methods

### Cell culture

HMSCs were purchased from Cambrex Corporation (East Rutherford, NJ, USA) and characterized as shown before [18]. Cells have been tested by Cambrex for purity by flow cytometry of surface markers (positive for CD105, CD166, CD29 and CD44; negative for CD14, CD34 and CD45). We have tested that SCP-1 is positive >99% for the stem cells markers CD105, CD73 and CD90 by FACS analysis (data not shown). The MSCGM BulletKit (Cambrex) was used as culture medium. Cells were cultivated in T-75 flasks (Nunclon, Nunc, Wiesbaden, Germany) in a humidified incubator at 5% CO_2_ and 37°C. Media were changed every 3–5 days and cells were trypsinized before confluence and counted by a hemocytometer. Cell morphology of untransduced, clonal and heterogeneous hTERT-transduced hMSCs (hTERT-hMSCs) was continuously observed and photographed using a phase contrast microscope (Axiovert, Carl Zeiss, Götingen, Germany).

### Cloning hTERT into the lentiviral vector

HTERT cDNA from pBABE-PURO plasmid was subcloned in pENTR11 plas-mid (Invitrogen, Karlsruhe, Germany) by blunt end SalI/ NotI ligation. The coding sequence of hTERT was then transferred from pENTR11 into pLenti6/V5-DEST by using LR Clonase according to manufacturer's protocol (Invitrogen). The correct sequence of the resulting pLenti6/V5-hTERT was confirmed by sequencing (Sequiserve, Vaterstetten, Germany).

### Lentivirus production and transduction of target cells

The ViraPower lentiviral expression system™ (Invitrogen) was used for lentivirus production and carried out following the manufacturer's instructions with minor adjustments [18]. The DNA-Lipofectamine™ complexes were added to a T-225 tissue culture flask containing 293FT cell suspension (23.76 × 10^6^ total cells). Forty-eight hours after transfection, the virus containing supernatant was harvested. The viral stocks were stored in aliquots at −80°C. Transduction of hMSCs was carried out with hTERT lentivirus (MOI: 5 **×** 10^4^) in the presence of 6 μg/ml polybrene (hexa-dimethrine bromide, Sigma, Munich, Germany). Successfully transduced cells were selected with blasticidin (10 μg/ml, Invitrogen) for 7 days. Single cells were picked under light microscopy at the 5^th^ passage and expanded into single-cell-picked clones (SCP). Among the 22 single-cell-picked clones, four clones (SCP-1, -9, -11 and -12) were maintained in cell culture and compared with heterogeneous hTERT-hMSCs and untrans-duced hMSCs.

### Detection of hTERT and tumour suppressor gene transcription by RT-PCR

To examine hTERT expression, RNA from SCP-1, SCP-9, SCP-11, SCP-12 and hTERT-hMSCs was isolated at 35–46 passages (with PDL between 31 and 54). To quantify tumour suppressor gene expression, RNA was extracted from SCP1, SCP9, SCP11, SCP12, heterogeneous hTERT-hMSCs and untransduced hMSCs at passage 8–13 (PDL 15–23), 35–46 (PDL 29–54) and 62–63 (PDL 72–86), respectively. Total RNA was extracted from each cell type performed with RNeasy® Mini Kit (Qiagen, Hilden Germany). For the reverse transcription (RT), 1 μg of RNA was converted into cDNA performed with AMV first-strand cDNA synthesis kit (Invitrogen) at 50°C for 50 min. The hTERT and tumour suppressor gene expression was detected by polymerase chain reaction (PCR) using *Taq* polymerase (Invitrogen), with the amplification of β-actin as control. The specific primers for hTERT, β-actin [19], retinoblastoma 1 [20], p53 [21], p21 [22], p16 [23], p14 [23] and PCR conditions are listed in Table 1. The products of the PCR were analysed by 1% agarose gel electrophoresis.

**Table 1:** Sequence of PCR primers and reaction conditions used in the experiments

Target gene	Primers	Annealing temp./time	Number of cycles	Reference
hTERT	Forward primer: 5′-CTACGGCGACATGGAGAAC-3′	55°C/30 sec.	35	
	Reverse primer: 5′-GACACTTCAGCCGCAAGAC-3′			
β-actin	Forward primer: 5′-GCACTCTTCCAGCCTTCC-3′	61°C/60 sec.	20	[19]
	Reverse primer: 5′-AGAAAGGGTGTAACGCAACTAAG-3′			
retinoblastoma 1	Forward primer: 5′-TTTCAGAAGGTCTGCCAACACCAA-3′	58°C/1 min.	35	[20]
	Reverse primer: 5′-GTGTCCACCAAGGTCaGAGATCC-3′			
p53	Forward primer: 5′-AAGGAAATTTGCGTGTGGAG-3′	58°C/45 sec.	32	[21]
	Reverse primer: 5′-TTCTGACGCACACCTATTGC-3′			
CDKN1A, p21	Forward primer: 5′-GAACTTCGACTTTGTCACCGAG-3′	60°C/30 sec.	30	[22]
	Reverse primer: 5′-CGTTTTCGACCCTGAGAGTCTC-3′			
p16INK4	Forward primer: 5′-CAACGCACCGAATAGTTACG-3′	56°C/60 sec.	35	[23]
	Reverse primer: 5′-AGCACCACCAGCGTGTC-3′			
p14ARF	Forward primer: 5′-GGGTTTTCGTGGTTCACATC-3′	57°C/60 sec.	25	[23]
	Reverse primer: 5′-CGCTGCCCATCATCATGAC-3′			

### Immunhistology

Immunohistological stainings were performed with untransduced hMSC at 7^th^ passage and SCP1 at 74^th^ passage (PDL 121). HMSCs were cultured on sterile glass cover slides and fixed with methanol at −20°C for 8 min. Immunostaining was performed with an anti-hTERT (348–358) rabbit poly-clonal antibody (CalBiochem, Germany) at a dilution of 1:10. Alexa Fluor 488 donkey anti-rabbit IgG was used as secondary antibody (Invitrogen) and nuclear counterstaining was performed with DAPI (Invitrogen). Negative controls for antibody were carried out on the same slide by omitting the primary antibody for both staining procedures.

### Telomerase activity assay

For quantitative determination of telomerase activity, the Telo TAGGG Telomerase PCR ELISA^Plus^ (Roche Diagnostics, Mannheim, Germany) was used according to the manufacturer's recommendations. Cell extracts from 2 × 10^5^ cells were resuspended in 200-μl lysis reagent. The telomeric repeat amplification protocol (TRAP) reaction was performed from 3-μl cell extract. Measurements were performed in triplets.

### Cell proliferation studies

Long-term cell growth *in vitro* was monitored by cell number count and cumulative population doubling level (PDL) calculation. Population doubling gained at each passage was determined using the formula: PD_(n/(n−1))_= (log(*N*_n_/*N*_n−1_))/log2 (n: passage; *N*: cell number). Cumulative PDL is the sum of population doublings.

### BrdU proliferation assay

The assay was performed according to the manufacturer's description (Cell proliferation ELISA, BrdU [colorimetric], Roche, Germany). Briefly, hTERT-hMSCs in young passages (14–15, PDL 24–26) and SCP-1 clone in young (14–15, PDL 29–31) and later (69–70, PDL 107–109) passages were used. The cells were seeded in 96-well dishes with a density of 3000 cells/well and were grown in complete culture media. After 12 hrs, the media was exchanged with complete culture media supplemented with 10 μM bro-modeoxyuridine (BrdU). The BrdU incorporation was measured after 24 hrs using microplate reader (Mikrotek Laborsysteme GmbH, Overat, Germany) at 450 nm with a reference wavelength of 690 nm. BrdU uptake was then calculated as a percentage to the young passage hTERT-hMSC cells. Two independent experiments were performed in triplicates.

### Senescence-associated β-galactosidase activity assay

The assay is based on histochemical staining for β-galactosidase activity at pH 6. Cells of untransduced, clonal and heterogeneous hTERT-hMSCs were cultured in 6-well plates and stained by Senescence Cells Histochemical Staining Kit (Sigma-Aldrich, Munich, Germany). Cells were washed with PBS and fixed. One millilitre of the staining mixture was added into each well. Plates were incubated at 37°C without CO_2_. Cells were examined after 12 hrs.

### Soft agar assay

The soft agar assay was performed by inoculating 5000 cells/6 well of clonal SCP-1 (passage 134, PDL 322) in 0.4% agar solution (37°C) and layered on top of 0.8% agar layer. One 6-well plate (30.000 cells total/clone) were seeded. Cells were incubated for 28 days at 37°C with 5% CO_2_. Plates were stained by 0.005% crystal violet for 1 h. Colonies were observed and photographed using a light microscope (Axiovert 100, Carl Zeiss). HT1080 cells were used as positive and untransduced hMSC as negative controls.

### Cytogenetics

Cytogenetic analysis was performed on SCP-1 (passage 92, PDL 175) SCP-11 (passage 89, PDL 127) and SCP-12 (passage 80, PDL 90). Harvesting and fixation followed standard protocols. Chromosome analysis was performed using the GTG-banding technique with a 400 bphs resolution. Fifteen metaphases captured by a CCD-camera were analysed and karyotyped using a karyotyping software. Chromosome identification and karyotype description were made in accordance with the International System for Chromosome Nomenclature [24].

### Fluorescence in situ hybridization (FISH)

To investigate whether the identified deletion found in cytogenetic analysis already pre-existed, two-colour FISH was performed on hTERT-hMSCs (passage 11, PDL 19) and hMSC (passage 12, PDL 18) using subtelomer-ic probes 16PTER and 16QTER (Abbott Diagnostics, Wiesbaden Germany). The 16PTER-probe was labelled in green (SpectrumGreen) and the 16QTER-probe in red (SpectrumOrange). Hybridization solution and probes were mixed 1:1 (followed manufacturer's protocols), dropped onto chromosome slides and covered. Co-denaturation of the slides was performed at 73°C for 2 min. followed by incubation at 37°C in a humidified chamber over night. After hybridization, the slides were washed following probe protocols and mounted in vectrashield mounting medium for fluorescence with DAPI (4′,6-diamino-2-phenylindole) (Linaris, Wertheim, Germany). Fluorescence signals were observed with a Zeiss fluorescence microscope. A total of 198 interphase nuclei were analysed.

### *In vivo* implantation assay

Twenty athymic nude mice (Harlan Winkelmann, Borchen, Germany) were divided into four groups with five mice in each group. A total of 1 **×** 10^6^ cells of each cell type (SCP-1, SCP-11, untransduced hMSCs and HT1080) were suspended in 250 μl of phosphate buffered saline (PBS) and injected subcutaneously over the right ribcage. Injections were performed under general anaesthesia with isoflurane. SCP-1 cells were used in 90^th^ passage (PDL 168), SCP-11 in 89^th^ passage (PDL 127). Mice were sacrificed after 8 weeks by CO_2_ overdose.

All procedures were performed according to German animal protection legislation and approved by the Government Committee of Upper Bavaria (file reference 07–07). Photographs were taken for macroscopic evaluation and dimensions of the tumour growth measured prior to dissection. Skin and underlying soft tissue of the relevant area were dissected. Specimens were fixated in 4% paraformaldehyde. Samples were processed *via* cryosectioning. Samples were fixated and tissues were infiltrated with sucrose solution (3 hrs in 5% sucrose, 3 hrs in 10% sucrose, 12 hrs 20% sucrose). Specimens were frozen in Tissue Freezing Medium (Jung, Germany). Serial cuts were prepared with a slice thickness of 12 μm. Representative slides of each animal were stained with haematoxylin and eosin and investigated for possible tumour growth.

### Differentiation and staining (von Kossa, Oil Red O, toluidine blue)

SCP-1 (47^th^ passage, PDL 57), SCP-11 (68^th^ passage, PDL 82) and hTERT-hMSCs (52^nd^ passage, PDL 57) were differentiated towards adipogenic, osteogenic and chondrogenic lineages [18]. Untransduced cells were used as a positive control. As a negative control both untransduced and transduced cells were cultured under identical conditions in standard medium (D-MEM, high glucose, glutamin, pyruvate) supplemented with 10% FBS and 1% Pen-Strep solution without differentiation supplements.

*In vitro* osteogenic differentiation of hMSCs was performed as previously published [18] using 100 nM dexamethasone, 10 mM β-glyc-erophosphate, 50 μM L-ascorbic acid-2-phosphate (Sigma). A total of 5 × 10^3^ cells/well were seeded in a 6-well plate. After 16 days, cells were assayed by von Kossa staining using a standard protocol.

Adipogenic differentiation was accomplished as previously published [18] by 1 μM dexamethasone, 0.2 mM indomethacin, 0.1 mg/ml insulin, 1 mM 3-isobutyl-1-methylxanthin (IBMX) (Sigma). The maintenance medium consists of 0.1 mg/ml insulin in standard medium. A total of 4 **×** 10^3^ cells/well were seeded in a 12-well plate. Stimulation was started when cells reached full confluency. Cells were grown for 5 days in induction medium, thereafter for 2 days in maintenance medium and then switched to induction medium again. After 16 days of stimulation, the cells were assayed by oil red O staining using a standard protocol.

Chondrogenic differentiation was achieved in aggregate cultures as previously published [18] with 100 nM dexamethasone, 1 mM pyruvate, 195 μm L-ascorbic acid-2-phosphate, 350 μM L-proline, 1.25% (v/v) insulin-transferrin-selenious acid mix (ITS, 100×), 5.35 μg/ml linolic acid, 1.25 mg/ml bovine serum albumine (BSA) (Sigma) and TGF-β_3_ (10 ng/ml; R&D Systems, Minneapolis, MN, USA). A total of 2.5 × 10^5^ cells were used per pellet. Sections of the size of 12 μm were cut with a cryostat vacutome HM 200 OM (Microm, Walldorf, Germany). Anionic sulphated proteoglycans were detected by toluidine blue metachromasia. Slices were stained in 1% toluidine blue solution (Sigma, Munich, Germany), 1% sodium tetraborate (Sigma, Munich, Germany).

### Statistical analysis

Statistical analysis was performed using SigmaPlot version 8 (SPSS, Munich, Germany) Significances were calculated using Student's t-test and chi-square test (FISH analysis). A value for *P* < 0.05 was considered significant.

## Results

### Ectopic expression of hTERT in hMSCs

**Fig. 1:**
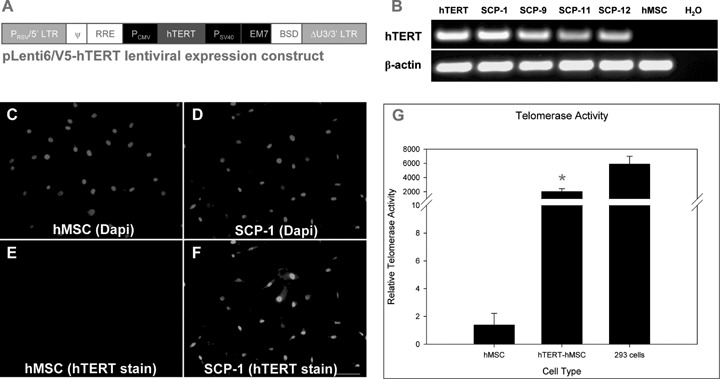
**(A)** Plasmid chart of pLenti6/V5-hTERT (lentiviral expression construct). **(B)** hTERT mRNA was detected in hTERT-transduced hMSCs. hTERT: heterogeneous hTERT-hMSCs, SCP: single-cell-picked hTERT-hMSCs. No hTERT expression was detected in untransduced hMSC. (**C-F**) HTERT localization in MSCs. Immunohistochemical staining of untransduced hMSC (**C, E**) and hTERT transduced hMSC/SCP-1 (**D, F**). Staining patterns and distribution of hTERT are shown in E and F, DAPI nuclear counterstaining in C and D. Bar **=** 100 μm. **(G)** Telomerase activity assay. Untransduced hMSC show only background level of relative telomerase activity. hTERT-hMSCs have a significant higher relative telomerase activity (*P < 0.001). 293 cells: positive control.

Lentiviruses containing the gene of hTERT were generated from the lentiviral expression construct pLenti6/V5-hTERT (Fig. 1A). After lentiviral transduction of hMSCs, four clones (SCP-1, -9, -11 and -12) were isolated by single cell picking. All clones and the heterogeneous hTERT-hMSCs expressed hTERT, while in untrans-duced hMSCs no hTERT expression was detected on mRNA level by PCR (Fig. 1B). In immunohistochemistry, hTERT protein was primarily located within the nucleus of the hTERT-expressing hMSCs (Fig. 1F). No hTERT-expression was detected in untransduced hMSCs (Fig. 1E). Consistent results were found with telomerase activity assay (Fig. 1G), where untransduced hMSCs only had background levels of telomerase activity, while hTERT-expressing hMSCs showed significantly higher levels comparable to 293 cells (positive control).

### Morphology, senescence and growth kinetics of hTERT-transduced hMSC cells

**Fig. 2:**
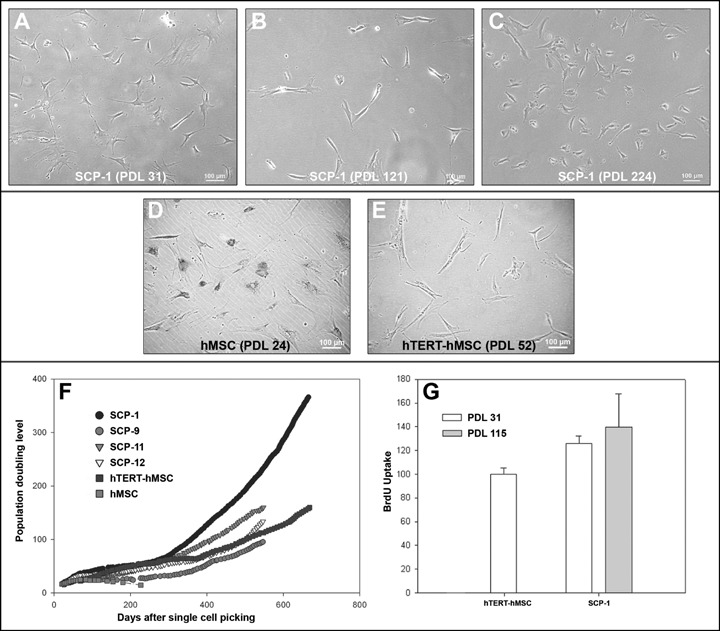
**(A-C)** Cell morphology at PDL 31, 121 and 224 of hMSC clone (SCP-1) ectopically expressing telomerase. **(D, E)** Senescence-associated **β**-galactosidase assay. Untransduced hMSCs show pronounced β-galactosidase activity at PDL 24. In contrast, hTERT-transduced hMSCs had no β-galactosidase activity even at higher passages. **(F)** Growth curve of untransduced hMSC (hMSCs), heterogeneous (hTERT-hMSCs) and single-cell-picked hMSC clones (SCP-1, -9, -11–12). (**G**) BrdU assay of heterogeneous hTERT-hMSCs and SCP-1 showed a significantly higher proliferation of SCP-1 *(P*= 0.00014 hTERT young [PDL 31]*versus* SCP-1 young passage [PDL 31]).

The morphology of clonal and heterogeneous hTERT-expressing hMSCs was monitored during the whole culture time. Normally, untransduced hMSC cultures exhibited heterogeneous morphological appearance with two predominant morphological pheno-types: small, round or spindle-shaped (RS cells) and flattened cells (FC cells) [25, 26]. In contrast, SCP-1 culture was much more homogeneous as it consisted mostly of RS cells (Fig. 2A and B). Furthermore, after more than 2 years no FC cells were detected in the SCP-1 culture (Fig. 2C). Additionally, untransduced hMSCs exhibited significant senescence-associated β-galactosi-dase activity around 24 PDL (Fig. 2D), while introducing hTERT into hMSCs resulted in an extension of their lifespan. This was reflected by the lack of detectable senescence-associated β-galac-tosidase activity even at later passages (PDL 52) (Fig. 2E).

Growth characteristics of four single-cell-picked hTERT-transduced hMSC clones (SCP-1, SCP-9, SCP-11 and SCP-12), the heterogeneous hTERT-hMSC and the untransduced hMSCs were monitored by calculating the population doubling level (PDL) for more than 2 years (Fig. 2F). As shown in Fig. 2F, there were three distinct growth periods of hTERT-expressing hMSCs. In the initial period, SCP-1, SCP-9, SCP-11, SCP-12 and heterogeneous hTERT-hMSCs had shown a PDL comparable to untransduced hMSCs (Fig. 2F). Then the hTERT-expressing clonal or heterogeneous hMSCs went into a growth plateau, while untransduced hMSCs became senescent. Finally, hTERT-expressing hMSC went into phase of rapid growth indicating a selection of faster growing cells. BrdU assay of heterogeneous (hTERT-hMSCs) and SCP-1 cells showed a significantly higher proliferation of SCP-1 clone *(P*= 0.00014 hTERT *versus* SCP-1 at PDL 31). This higher proliferation rate was maintained during extended culture periods (Fig. 2G).

### Evaluation of potential neoplastic transformation of hTERT-transduced hMSCs

**Fig. 3:**
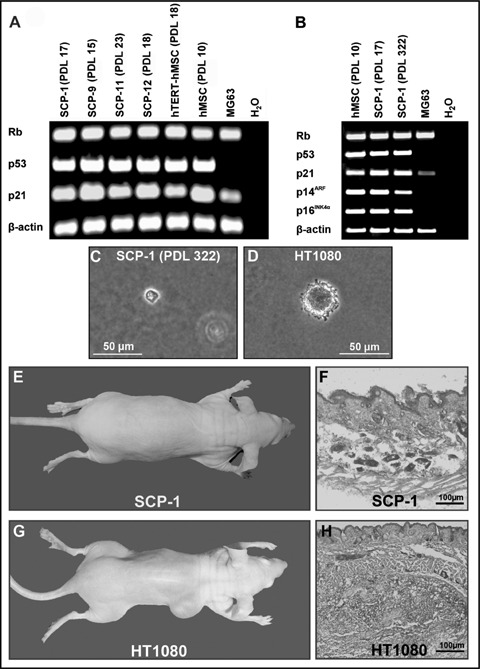
**(A)** Expression of tumour suppressor genes. There is no relevant change in tumour suppressor gene (Rb, p53 and p21) expression at different time points in heterogeneous (hTERT-hMSCs) and single-cell-picked hMSCs (SCP) in PCR analysis. MG63 (positive control) showed a marked down-regulation of p53 and p21. **(B)** Expression of p53, p21, p14 and p16 in SCP-1 (PDL 17 and 322). There was no significant down-regulation of these genes even after almost 2 years in culture. **(C, D)** Soft agar assay. HTERT-transduced hMSCs did show contact inhibition, while the tumour cell line HT1080 formed colonies. **(E–H)***in vivo* tumour formation. HTERT-transduced hMSCs (here shown SCP-1) did not show macroscopic **(D)** and microscopic (**E**, haematoxylin and eosin stain) after 8 weeks. In contrast, HT1080 formed macroscopically visible tumours within 1 week **(F)**. Haematoxylin and eosin stain revealed a characteristic tumour growth.

The expression profile of retinoblastoma tumour suppressor protein (Rb) and p53 was investigated as suggested in [27]. In addition, we analysed the expression of p21, a known key regulator of p53. Time-course analysis of Rb, p53 and p21 expression (Fig. 3A) revealed that in none of the hTERT-transduced hMSCs these key tumour suppressors were down-regulated, suggesting the growth of hTERT-expressing hMSCs was still under control of these ‘gatekeepers’. In contrast, the osteosarcoma cell line MG63 had no detectable levels of p53 and showed down-regulation of p21 (Fig. 3A).

Since a deletion of the Ink4a/ARF gene locus has been described after ectopic hTERT using retroviral vectors, we have analysed the two gene products: p14^AFR^ and p16^Ink4a^ (Fig. 3B). p14^AFR^ blocks MDM2-induced p53 degradation and p16^Ink4a^ inhibits CDK4(6) which in turn prevents cells with functional Rb from entering S phase. Using RT-PCR, we found that p14^AFR^ and p16^Ink4a^ expression was preserved even in passage 138 (PDL 322) of SCP-1, but deleted in the osteosarcoma cell line MG63. Taken together, our results support the idea that ectopic expression of hTERT in hMSCs did not cause malignant transformation by deletion of the Ink4a/ARF gene locus.

Next, we performed a functional *in vitro* assay to evaluate the neoplastic transformation potential of hTERT-expressing hMSCs. Due to a loss of anchorage dependency, which is a key feature of neoplastic transformation, tumour cells like HT1080 usually form colonies in soft agar assay (Fig. 3D). In contrast, SCP-1 did not form colonies within 28 days (Fig. 3C), indicating that cell proliferation of these clones was still controlled anchor-dependant.

Furthermore, we tested whether hTERT-transformed hMSCs show tumour formation *in vivo*. Therefore, two clones (SCP-1 and -11), untransduced hMSCs and the tumour cell line HT1080 were injected subcutaneously into five nude mice each. None of the animals in the SCP-1, SCP-11 and hMSC groups developed a macro-scopically detectable tumour by 8 weeks after implantation (Fig. 3E). Four of five animals in the HT1080 group were sacrificed 10 days after injection because of extensive tumour growth at the site of injection (Fig. 3G). The fifth animal had a macroscopically visible tumour upon explantation after 8 weeks. Histological evaluation of the injection sites confirmed the macroscopic findings (Fig. 3G and H). In all the animals from the SCP-1, SCP-11 and hMSC groups, a normal structure of the skin and subcutaneous tissue was observed. At the same time, extensive tumour growth was observed at the site of injection of all the animals that had received HT1080 cells.

### Karyotyping hTERT-transduced hMSCs

**Fig. 4:**
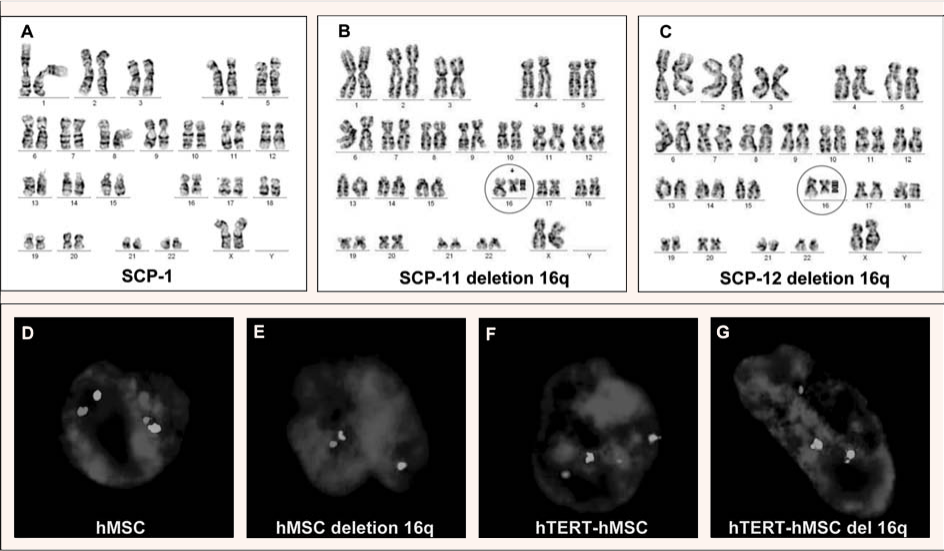
**(A–C)** Karyotyping revealed no change in hTERT-transduced SCP-1 clone (passage 92, PDL 175), while SCP-11 (passage 89, PDL 127) and SCP-12 (passage 80, PDL 90) showed identical deletions in the long arm of chromosome 16 (16q). **(D–G)** FISH analysis revealed that these deletions already existed in younger passages of untransduced and hTERT-transduced hMSCs and significantly accumulated with time (*P* < 0.018).

The hTERT-transduced hMSC clones SCP-1, SCP-11 and SCP-12 were found to be diploid with 46 chromosomes. There was no chromosomal aberration seen in SCP-1 cell clones (Fig. 4A). In contrast, SCP-11 and SCP-12 showed an identical deletion of the long arm of chromosome 16 (Fig. 4B and C). In order to exclude a chromosomal aberration related to lentiviral gene transfer, we went back to the original untransduced hMSCs (passage 12) and young hTERT-transduced cells (passage 11 after transduction) from the same donor. Fluorescence in situ hybridization (FISH) revealed that these deletions have already been present in the donor cells before lentiviral transduction. In the sub-telomer analysis of untransduced hMSCs, 5 of 194 (2.6%) interphase nuclei contained only one signal for the region 16qter (Fig. 4D and E). In heterogeneous hTERT-expressing hMSCs, 17 of 198 (8.6%) interphase nuclei lacked one region of 16qter (Fig. 4F and G). These results not only indicate that the chromosomal aberration already existed in donor hMSCs before lentiviral transduction, but significantly accumulated with continuous cell culture *(P* < 0.018). Nevertheless, the hTERT-expressing SCP-1 clone exhibited a regular karyotype (Fig. 4A).

### *In vitro* differentiation capacity of hTERT-expressing hMSCs

**Fig. 5:**
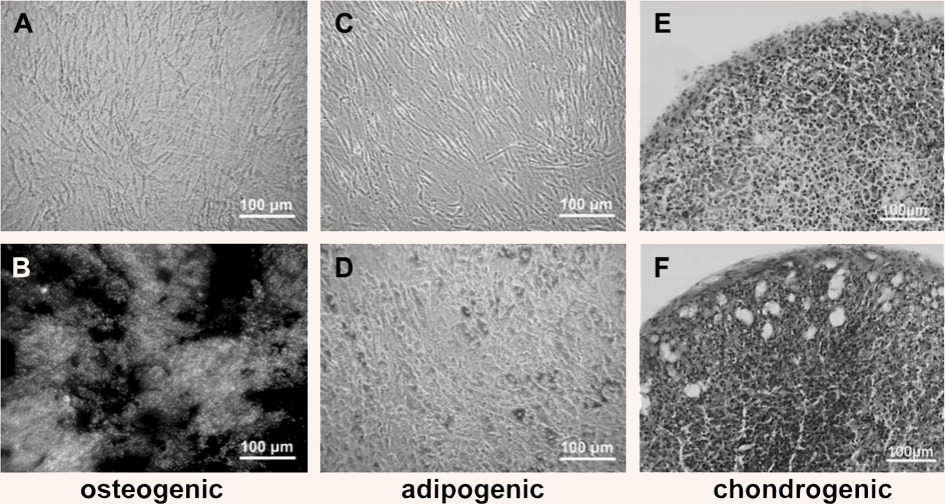
Differentiation assay. HTERT-transduced hMSCs (SCP-1) maintained their stem cell character and were still capable to differentiate into the osteogenic **(B)**, adipogenic **(D)** and chondrogenic **(F)** lineages. **(A, C, E)** Undifferentiated control cells.

SCP-1 (47^th^ passage, PDL 57), SCP11 (68^th^ passage, PDL 82) and hTERT-hMSCs (52^nd^ passage, PDL 57) were differentiated into three different mesodermal lineages (osteoblasts, adipocytes and chondrocytes). For late passages, we have proved differentiation capacity into osteogenic (PDL 146) and adipogenic (PDL 210) lineages. Evaluation was carried out by histology. Additionally, PCR was performed against transcription factors Osterix and PPAR-γ (data not shown). These cells maintained their differentiation capacity during long-term culture. After osteogenic differentiation, von Kossa staining was strongly positive (Fig. 5B). After adipogenic differentiation, clones stained strongly positive for intracellular lipid droplets using Oil red O staining (Fig. 5D), while extracellular proteoglycans were stained with toluidine blue after chondrogenic differentiation (Fig. 5F). Lineage specific staining was low or negative in controls without induction (Fig. 5A, C and E). These results indicate that hTERT-expressing hMSCs maintained their stem cell character during extended culture periods.

## Discussion

The regeneration of damaged and diseased tissue is the principal goal of tissue engineering and cell-based gene therapy and the availability of constituent cells is an essential requirement. Differentiated cells that replace the damaged or diseased tissue are desired, but their cell number is limited and their propagation leads often to dedifferentiation *in vitro.* Therefore, progenitor cells like MCSs, which can be propagated *ex vivo,* have been considered as an attractive alternative cell source for tissue engineering and cell-based gene therapy. They can be readily isolated from bone marrow aspirations and expanded *ex vivo.* Pioneering work by Friedenstein *et al.* has shown that bone MSCs can be differentiated into cells of osteogenic, adipogenic and chondrogenic lineages [28]. Animal studies strongly suggest that implanted hMSCs differentiate in a tissue-specific manner and are able to regenerate mesenchymal tissue such as muscle, bone and cartilage. But studies have shown that the clinical use of hMSCs may be limited by senescence-associated growth arrest under current *in vitro* culture conditions, a phenomenon termed replicative senescence [3]. Our results go in line with others that have found that senescence leads to growth arrest of hMSCs and eventually cell death after no more than 24–40 population doublings [4, 6, 29].

Replicative senescence is generally associated with telomere length shortening of chromosomes, which occurs with each cell division. Telomere length can be maintained by a catalytic subunit with reverse transcriptase activity (TERT). Although TERT is expressed in the stem cell compartment of several tissues [30], our results are consistent with the literature that cultured hMSCs exhibit no telomerase activity, suggesting that hMSCs lose telomerase expression during commonly used *in vitro* culture conditions [31, 32]. Telomere length in cultured hMSCs is similar to other somatic cell types [9] and *in vitro* expansion of hMSC results in continuous telomere shortening, which eventually contributes to hMSC senescence *in vitro*. The lack of telomerase activity in hMSCs can be overcome by ectopic expression of hTERT. Using this approach, stable expression of hTERT prevents replicative senescence in hMSCs [10, 12, 14, 33–35], with an extension of lifespan for more than 3 years [36]. These findings are consistent with our results that stable hTERT expression causes a continuous proliferation of hMSCs with a lack of senescence-associated β-galactosidase staining, while untransduced cells go into senescence with growth arrest.

Usually, long-term *in vitro* culture of hMSCs results in impaired differentiation capacity [37]. Remarkably, we and others have found that extended culture of hTERT-expressing hMSCs did not influence their stem cell character. The cells retained their ability to undergo osteogenic, adipogenic and chondrogenic differentiation. In fact, it was shown that MSCs overexpressing hTERT exhibit an increased osteogenic differentiation potential [14], while telomerase deficiency impairs differentiation of hMSCs [38].

Our results go in line with others who have found that hMSCs overexpressing hTERT exhibit an accelerated proliferation with advanced culture time. Adult stem cells from telomerase-deficient mice (*Terc^−/−^*) show a decreased proliferation potential [39, 40], while TERT-overexpression even in the absence of changes in telomere length promotes stem cell proliferation *in vitro*[41, 42]. These observations suggest an additional effect of telomerase on stem cell proliferation, which can bypass telomerase complex formation and telomere lengthening. The mechanisms by which telomerase overex-pression causes stem cell proliferation are currently unknown. Our finding that ectopically hTERT-expressing hMSCs have an accelerated proliferation with advanced culture time may not represent a deregulation of cell division, but rather a self-selection process of faster proliferating cells. Consistent with this hypothesis, we found with prolonged culture time an increased percentage of morphologically smaller cells. These cells have a similar morphology, growth pattern and differentiation potential comparable to a subset of hMSCs, which have been previously described as rapid self-renewing (RS) cells [25, 26]. These cells have a small, more round-shaped morphology, a high rate of replication and an enhanced potential for multi-lineage differentiation [25, 43]. Therefore, ectopic expression of hTERT in hMSCs may not only prolong the lifespan of hMSCs, but also favour the selection of a subset of rapidly self-renewing hMSCs with a higher potential for cell and gene therapy.

Several gene transfer methods have been explored in MSCs [44, 45]. For most gene therapy applications, long-term gene expression with stable integration of the transgene is necessary. Although in the past most studies have used retroviruses to ectopically express hTERT in hMSCs [10, 14], more recent results show that lentiviruses have consistently higher transduction rates in hMSCs with long-term gene expression [46].

We have previously shown that lentiviral gene transfer results in high levels of transgene expression without losing the differentiation potential of hMSCs [18]. Until now there is only one study using lentiviral gene transfer for ectopic expression of hTERT, but researches failed to prolong the lifespan of mesenchymal progenitor cells derived from human placenta [47]. We have now for the first time generated an MSC line with ectopic hTERT expression using lentiviral gene transfer. These cells show persistent hTERT expression even at later passages with significantly increased telomerase activity.

Long-term culture of hTERT-transduced adult MSCs using retro-virus resulted in neoplastic transformation [15, 16]. In our experiments using lentiviral gene transfer, we did not find malignant transformation of hTERT-transformed hMSCs. Both heterogeneous and single-cell-picked clones did not lose contact inhibition *in vitro* and did not form tumours *in vivo*, despite the fact that clones with a preexisting deletion in chromosome 16 were taken along. Insertional mutagenesis has been a limitation of retroviral gene transfer. Since oncogenesis occurred at unexpected high frequency in the X-SCID gene therapy trail and lentiviral vectors have high similarity to gamma-retroviral vectors, it has been a major concern that lentiviral vectors may also cause insertional mutagenesis. But so far, no adverse events have been reported upon transplantation of lentivirus vector-transduced cells. The only human gene therapy trial using third-generation lentiviral vectors started in July 2003 for treatment of HIV patients. To date, none of the patients in the clinical trial had experienced any adverse events due to the treatment [48]. Furthermore, a recent study which also used a third-generation lentiviral vector demonstrated that lentiviral vector transduction even at high integration loads did not accelerate any tumourgenesis in a tumour-prone mouse model. In contrast, in this study retroviral vector transduction triggered a dose-dependent acceleration of tumour onset dependent on vector LTR activity and integration at known proto-oncogenes and cell cycle genes [49]. The major reason for the low genotoxicity of lentiviral vectors may be the lack of transcriptionally active LTRs. To date, all available data suggest that lentiviral vectors are save vehicles for *ex vivo* gene therapy.

Neoplastic transformation of hTERT-immortalized hMSCs generated by retroviral gene transfer occurred after long-term culture. Serakinci *et al.*found alterations in tumour-related genes such as KRAS, NRAS, p14^AFR^/p16^Ink4a^ and DBCCR1 [16]. Deletion of the p14^AFR^/p16^Ink4a^ gene locus occured as early as 95 and 123 PDL, depending on the cell clone. SCP-1 did not loose the gene locus even after 322 PDL. The mechanisms by which retroviral hTERT-transduced hMSCs underwent neoplastic transformation are not fully understood. Although long-term *in vitro* culture of hMSCs by itself may not cause malignant transformation [50], *in vivo* the persistence and continued mitotic activity of stem cells throughout life makes these cells a potential reservoir for the accumulation of oncogenic mutations. There is evidence that some cancers arise from transformed stem cells [51, 52].

Cell proliferation and DNA replication are under surveillance of two well-established pathways, Rb and p53, that act as ‘gatekeepers' to promote senescence and cell death of transformed cells [27]. The p53 pathway is regulated in part by p21 expression. The Rb pathway has an important role in cell cycle control. We found in a time-course analysis of Rb, p21 and p53 expression that these key tumour suppressors were not down-regulated, suggesting the growth of hTERT-transduced hMSCs were still under control of these ‘gatekeepers’, which was consistent with the result of other authors [53, 54].

Transformation may have evolved from spontaneous chromosomal aberrations in prolonged culture with multiple cell divisions and may not have been directly related to ectopic hTERT expression. This hypothesis is supported by the finding that spontaneous accumulation of chromosomal abnormalities and malignant transformation occurred after numerous passages even in untransduced murine bone marrow-derived MSCs [55]. On the other hand, mathematical modelling indicates that rather a selective growth of cells with mutations in tumour suppressor genes is the driving force in the development of most human tumours, than an increased mutation rate. Spontaneous transformation in cultured cells is efficiently evoked by progressive selection under prolonged contact inhibition at high population density or during multiplication at low population density in suboptimal concentrations or types of serum [56]. Therefore, malignant transformation of hTERT-transduced hMSCs might have been the result of progressive selection of malignant cells under particular culture conditions. Since germline cells, embryonic stem cells and some active somatic cells express hTERT *in vivo* and many different cell types have been immortalized by hTERT without malignant transformation, hTERT is not considered an oncogene [17]. Therefore, hTERT may rather support the selection of pre-existing mutations. This hypothesis is supported by our finding that pre-existing mutations like the deletions on chromosome 16 accumulated with prolonged culture time. Furthermore, unlike lentiviral vectors that show low tumourgenesis, retroviral vectors are known to trigger a dose-dependant tumour acceleration [57] and may, therefore, further increase the risk of malignant transformation of hMSCs. These findings also emphasize the need for caution in the use of hTERT-immortalized hMSCs in cell and gene therapy applications and the need to monitor for genetic instability and accumulation of mutations.

In summary, we report for the first time an hMSC clone successfully immortalized by ectopic expression of hTERT using lentiviral gene transfer. The immortalized clonal hMSCs revealed no change of karyotype with no signs of malignant transformation *in vitro* and *in vivo*, making them an attractive candidate for cell and gene therapy applications. Nevertheless, due to a potential risk of selecting pre-existing tumour cells, careful screening for mutations and malignant transformation is mandatory.
